# *Aristolochia ferox*, a new species of subg. *Siphisia* (Aristolochiaceae) from Guangxi, China

**DOI:** 10.3897/phytokeys.273.169468

**Published:** 2026-04-17

**Authors:** Yifan Wang, Shuai Liao, Sven Landrein, Khang Sinh Nguyen, Joyce G. Onyenedum, Zirui Guo

**Affiliations:** 1 Department of Environmental Studies, New York University, New York, NY 10012, USA Department of Environmental Studies, New York University New York United States of America; 2 Key Laboratory of East China Plant Conservation and Utilization, National Forestry and Grassland Administration, Shanghai Chenshan Botanical Garden, Shanghai 201602, China Institute of Biology, Vietnam Academy of Science and Technology Hanoi Vietnam; 3 Kadoorie Farm and Botanic Garden (KFBG) Corporation, Hong Kong S.A.R., China Shanghai Chenshan Botanical Garden Shanghai China; 4 Institute of Biology, Vietnam Academy of Science and Technology, Hanoi 10072, Vietnam Kadoorie Farm and Botanic Garden (KFBG) Corporation Hong Kong Hong Kong; 5 Kunming Sanqian Horticulture Technology Co., Ltd., Kunming 650500, Yunnan, China Kunming Sanqian Horticulture Technology Co., Ltd. Kunming China

**Keywords:** Critically Endangered, endemics, liana, morphology, perianth, plant taxonomy

## Abstract

*Aristolochia ferox* is described as a new species endemic to the Jiuwanshan Mountains, northern Guangxi, China. It is morphologically similar to *A. championii*, *A. quangbinhensis*, *I. haimingii* and *A. fangchi* in vegetative characters but is readily distinguished by its floral traits, particularly the adaxial calyx limb, which is wholly dark purple and muricate with coarse, fleshy, conical outgrowths. The new species is restricted to a very small geographic range. Field surveys indicate that it is extremely rare, with very small extant populations, and is potentially threatened by ethnobotanical harvesting. Accordingly, it is preliminarily assessed as Critically Endangered (CR C2a(i), D) under the IUCN Red List criteria.

## Introduction

The genus *Aristolochia* L., long recognized for its extraordinary floral architecture and ecological diversity, is the most species-rich lineage of Aristolochiaceae, comprising more than 500 taxa worldwide (Wagner et al. 2014; Wanke et al. 2017). Its characteristic tubular, connate calyx has given rise to the vernacular name “pipevine.” According to the latest APG IV system and recent taxonomic treatments, *Aristolochia* s.l is composed of three subgenera: i.e. subg. *Aristolochia*, subg. *Pararistolochia* (Hutch. & Dalziel) Schmidt, and subg. *Siphisia* (Duch.) Schmidt (González and Stevenson 2002; Wanke et al. 2006; Buchwalder et al. 2014; Ohi-Toma and Murata 2016).

For *Aristolochia* subg. *Siphisia*, phylogenetic analyses consistently recover it as monophyletic and as the earliest-diverging branch of *Aristolochia*, sister to a clade comprising subg. *Aristolochia* and subg. *Pararistolochia* (Ohi-Toma et al. 2006; Wanke et al. 2017; Zhu et al. 2019; Jost and Wanke 2024; Hu et al. 2025). In recent years, subg. *Siphisia* has also experienced a rapid pace of species discovery and taxonomic revision, particularly in southern China and northern Vietnam (Do et al. 2021; Zhang et al. 2024; Guo et al. 2025; Wang et al. 2025). Many of these newly described taxa are narrowly endemic, often restricted to habitats that are difficult to access (Guo et al. 2025), which may partly explain why, despite their conspicuous floral morphology, they have only recently come to light.

During fieldwork in the Jiuwanshan Mountains, Rongshui Miao Autonomous County, Liuzhou City, Guangxi Zhuang Autonomous Region, China, we encountered a *Aristolochia* species flowering on both old stems and in leaf axils. Preliminary field identification suggested that it belongs to subg. *Siphisia*. This taxon exhibits a unique and previously undocumented floral morphology, which was consistently observed across multiple populations. The perianth bears a calyx limb wholly dark purple on the adaxial side and muricate with fleshy, prickly, conical outgrowths. This combination of morphological characters clearly distinguishes the taxon from all described species of subg. *Siphisia*. We therefore describe it here as a new species.

## Materials and methods

### Material sampling

Field surveys were conducted in May, June, August, and November 2024; and in August and December 2025, in Rongshui County, Liuzhou City, Guangxi Zhuang Autonomous Region, China. During these survey periods, intensive investigations were undertaken in the townships of Sirong, Sanfang, Wangdong, Anchui, and Xiangfen to document the distribution and population status of the species.

The new species were collected both as voucher specimens and as living material. Duplicate type specimens were deposited in BAZI, CSH, HK, IBK, IBSC, KUN, and PE. Living plants were transplanted and maintained for ex-situ conservation at the Botany Garden of Guilin, Guangxi Institute of Botany.

### Taxonomic and morphological assessment

In some taxonomic treatments of *Aristolochia*, the lineage traditionally recognized as subg. *Siphisia* has been elevated to generic rank as *Isotrema* Raf. (Zhu et al. 2019), and several species have subsequently been described under that name, resulting in two parallel nomenclatural frameworks for the same clade (Huang et al. 2022). In the present study, we retain *Siphisia* at subgeneric rank within *Aristolochia*. No taxonomic revision of species previously published under *Isotrema* is undertaken here; such taxa are cited under their original combinations. Accordingly, both generic names are retained where applicable.

Morphological characters were assessed with reference to original species descriptions and published taxonomic keys for *Aristolochia* (Do et al. 2015; Zhu and Ma 2022). Measurements and observations were obtained from both examined voucher specimens and fresh living material. For living material, flowering individuals were sampled and longitudinally dissected to observe internal characters of the perianth tube and gynostemium.

Living material examined and photographed in this study included the new species observed during fieldwork on 15 July 2023 (Renping Jia and Zirui Guo *yw00140*); cultivated individuals of *Aristolochia championii* Merr. & Chun on 21 March 2024 at the Kadoorie Farm and Botanic Garden with assistance from the Department of Flora Conservation (accession not applicable); *Aristolochia quangbinhensis* T.V.Do observed during fieldwork on 7 March 2024 (Khang Sinh Nguyen et al. *QT-DR 12*); *Isotrema haimingii* Y.S.Huang & Yan Liu examined on 28 June 2024 from cultivated material at the Botany Garden of Guilin (Yusong Huang *Y3310*); and *Aristolochia fangchi* Y.C.Wu ex L.D.Chow & S.M.Hwang examined on 24 May 2024 from cultivated material at the Ningbo Botanical Garden (accession not applicable). Selection and analysis of diagnostic morphological characters followed recent taxonomic treatments adopted by Guo et al. (2025) and Wang et al. (2025). Complete specimen information is provided in Appendix 1.

### Conservation status assessment

The conservation status assessment followed the IUCN Red List Categories and Criteria (IUCN 2012) and the Guidelines for Using the IUCN Red List Categories and Criteria (IUCN 2024). The Extent of Occurrence (EOO) and Area of Occupancy (AOO) were calculated using GeoCAT (Bachman et al. 2011), based on documented localities and population records. Exact locality coordinates are withheld for conservation reasons but can be provided by the corresponding authors upon request for bona fide research purposes.

## Taxonomy

### Description

#### Aristolochia ferox

Taxon classification

Plantae

PiperalesAristolochiaceae

Y.Fan Wang, Z.R.Guo & J.G.Onyenedum sp. nov.

D8625E01-05C7-5956-B233-0D5C9419736C

urn:lsid:ipni.org:names:77378733-1

Figs 1, 2

##### Type.

China. • Guangxi: Liuzhou City, Rongshui Miao Autonomous County, Anchui Township, 1175 m a.s.l., 15 Jul 2023, *Renping Jia & Zirui Guo yw00140* (holotype: IBK! [00473343]; isotypes: BAZI!, CSH!, HK!, IBSC!, KUN!, PE!).

**Figure 1.**
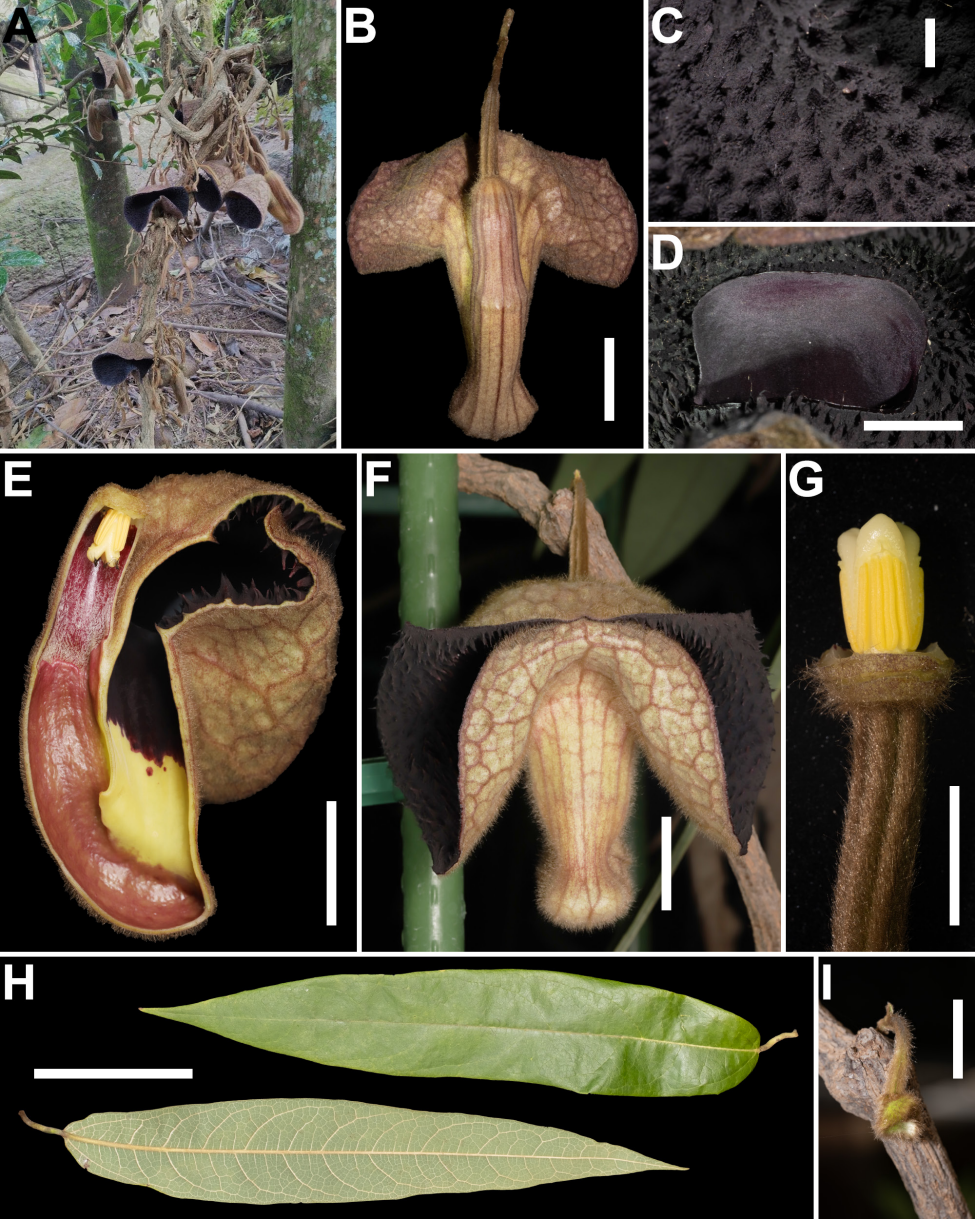
Morphological features of *Aristolochia ferox* Y.Fan Wang, Z.R.Guo & J.G.Onyenedum, sp. nov. **A**. Habit in situ, flowering on old stem; **B**. Abaxial view of flower; **C**. Adaxial surface of calyx limb densely covered with coarse, dark purple, fleshy conical papillae; **D**. View of calyx throat; **E**. Longitudinal dissection of flower showing internal adaxial tube structure; **F**. Frontal view of flower; the lower part of the limb is markedly reflexed upwards, partially obscuring the throat; **G**. Gynostemium in staminate phase: stigma retracted and non-receptive; anthers dehiscing pollen; **H**. Adaxial and abaxial surfaces of the leaf; **I**. Early sprouting of flower buds from lignified nodes, showing cauliflory; abaxial surface of immature flowers densely covered with rusty villous indumentum. Scale bars: 2.0 cm (**B, E, F, I**); 5.0 mm (**C**); 1 cm (**D, G**); 10 cm (**H**).

**Figure 2.**
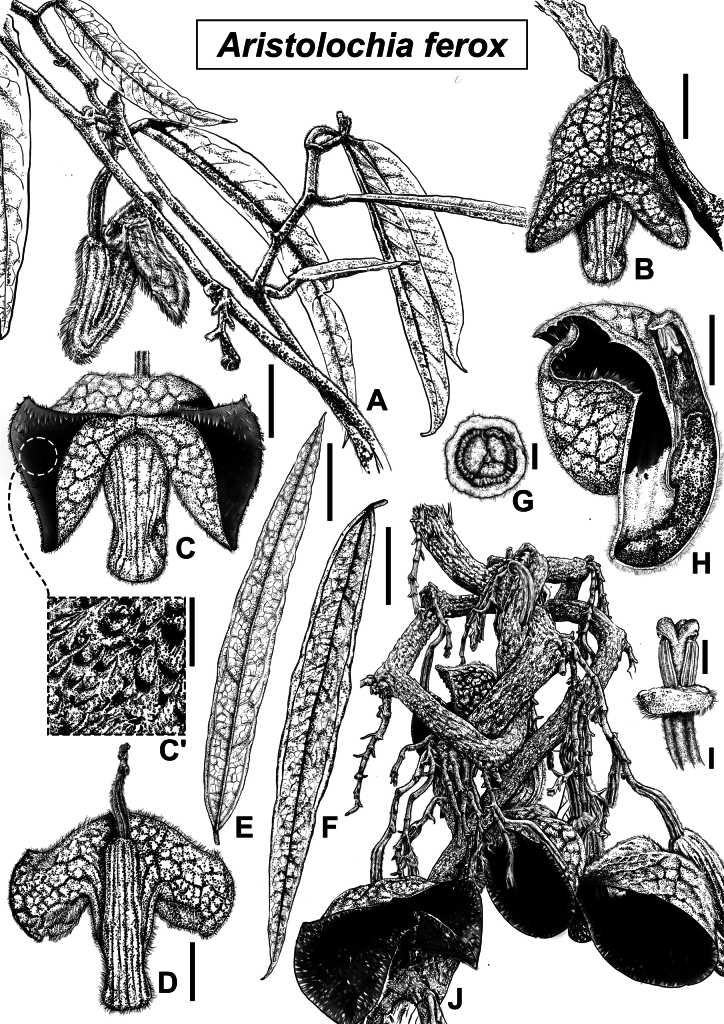
Line drawing of *Aristolochia ferox*. **A**. Young branch bearing leaves and axillary inflorescence; **B**. Floral bud at anthesis; **C**. Flower, frontal view; **C'**. Enlarged detail of calyx limb, enlarged; **D**. Rear view of flower; **E**. Abaxial surface of leaf; **F**. Adaxial surface of leaf; **G**. Top view of gynostemium showing three-lobed stigma; **H**. Longitudinal dissection of flower; **I**. Lateral view of gynostemium; **J**. Basal portion of mature stem with cauliflorous inflorescences emerging from nodal regions. Line drawing by Ms. Yushan Cai. Scale bars: 2 cm (**B, C, D, H**); 5.0 mm (**C'**); 5 cm (**E, F**); 2.0 mm (**G**); 5.0 mm (**I**).

##### Diagnosis.

**Figure 3.**
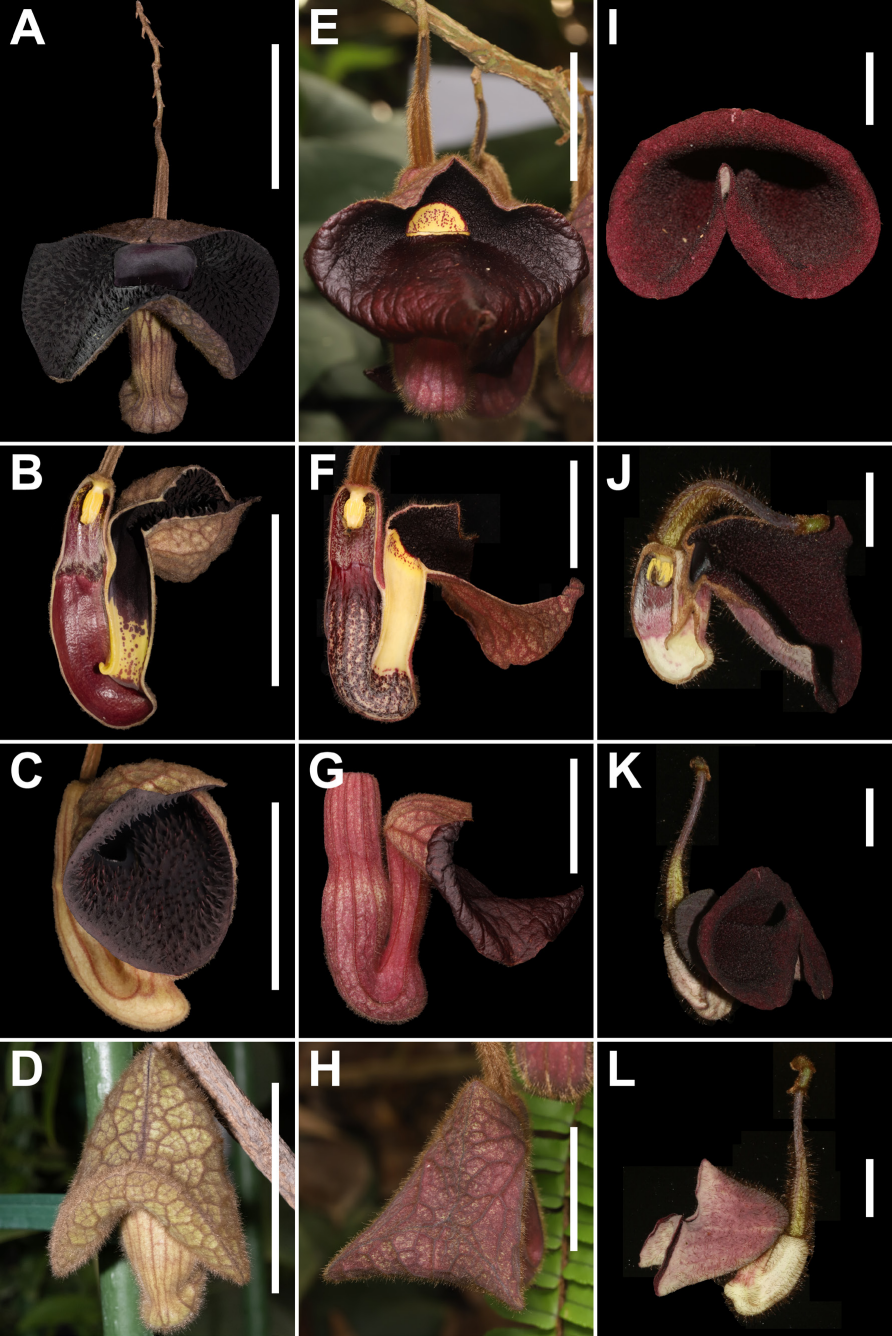
Comparative floral morphology of *Aristolochia ferox* (**A–D**), *A. championii* (**E–H**), and *A. quangbinhensis* (**I–L**). **A, E, I**. Frontal view; **B, F, J**. Longitudinal section; **C, G, K**. Lateral view; **D, H, L**. Buds at pre-anthesis. Images **A–C** are provided by Dr. Yusong Huang. Scale bars: 5.0 cm (**A–D**); 2.5 cm (**E–H**); 1.0 cm (**I–L**).

*Aristolochia ferox* is most similar to *A. championii* in its woody lianous habit, narrowly lanceolate to subulate leaves with rounded to truncate bases, and a large, campanulate calyx limb, but differs by the calyx limb adaxially entirely dark purple and densely muricate with coarse, fleshy, conical outgrowths, with the lower margin revolute upward, strongly incurved and connivent, auriform in frontal view (vs. reddish-brown, colliculate only around the throat, the lower margin obliquely deflexed), and by the throat deep dark purple, elliptic (vs. yellow with purplish-red flecks, semicircular to arch-shaped). This distinctive floral syndrome readily separates the new species from other superficially similar species, including *A. quangbinhensis*, *A. fangchi*, and *I. haimingii*, which lack this morphology. Detailed morphological comparisons among *A. ferox* and its closely related congeners are provided in Fig. 3, Suppl. material 1: fig. S1, and Table 1.

**Table 1.** Comparison between *A. ferox* and its morphologically closest relatives.

Characters	*A. ferox* sp. nov.	*A. championii*	*A. quangbinhensis*	*I. haimingii*	*A. fangchi*
Distribution	Rongshui County and Huangjiang County, Guangxi, China	Hong Kong and eastern Guangdong, China	Central Vietnam	Jinxiu County, Guangxi, China	Guangdong and Guangxi, China; northern Vietnam
Petiole	1.5–2.5 cm, pubescent	1–2 cm, densely rusty-villous	1.5–3 cm, densely yellow-brown villous	1–2 cm, densely yellowish-brown-villous	1–4 cm, densely rusty villous
Lamina	13–36 × 3–6 cm, narrowly lanceolate to linear-lanceolate	12–25 × 2–5 cm, lanceolate to linear-lanceolate	6–14 × 3–6 cm, elliptic to oblong-elliptic	12–25 × 3–6 cm, lanceolate to elliptic-lanceolate	6–12 × 3–4 cm, oblong or ovate-oblong, rarely lanceolate
Leaf apex	Acuminate	Acuminate	Acute or obtuse	Narrowly acuminate	Obtuse or mucronate
Leaf base	Rounded or truncate	Rounded or truncate	Narrowly auriculate	Rounded or shallowly cordate	Rounded or truncate
Peduncle	8–15 cm, densely rusty-villous	3–4 cm, rusty-villous	1.5–2 cm, densely villous	2.5–4 cm, densely yellowish-brown villous	5–7 cm, rusty-villous
Limb (adaxial)	5–6 × 7.5–10 cm, adaxial surface entirely black, muricate with coarse fleshy conical outgrowths; limb margin strongly revolute basally, lower edge curving upward to meet upper edge and concealing throat in frontal view, forming distinctly auriform, connivent aspect	3–5 × 4–6 cm; adaxial surface reddish-brown, colliculate around the throat, otherwise flat and waxy; limb bell-shaped, with the basal portion elongated and extended downward	2–3 × 3–5 cm, adaxial surface entirely verrucose, vinaceous in color; limb with the basal margin reflexed upward, producing a binocular-like aspect in frontal view	3–5 × 5.5–6.5 cm; adaxial surface yellowish-green, upper portion with dark purple mural-like stripes, lower portion with mauve stripes; limb discoid-shaped; margin shallowly 3-lobed	3–5 × 5–7 cm; adaxial surface purplish brown, with conspicuous dark purple reticulate or radiating venation and irregular white bands or dots; limb discoid; margin shallowly 3-lobed; surface gently undulate and velvety
Throat	1.8–3.0 × 1.5–2.0 cm, elliptic, velvety, black	1.2–1.8 × 1–1.5 cm, semicircular to arch-shaped, yellow with purplish-red flecks	0.6–0.8 × 0.3–0.4 cm, annular, dark violet	1.0–1.4 × 1.4–1.6 cm, suborbicular, velvety, yellowish-green or mauve, with dark purple stripes	1.2–1.8 × 1.6–2.0 cm, velvety orbicular, white or pale pinkish,

##### Description.

Woody liana 6–8 m long. Root system tuberous, composed of fusiform to globose segments constricted at intervals, forming a distinctly moniliform underground structure. Stems terete; young stems densely rusty-golden villous; older stems lignified, subcylindrical, bark finely fissured. Leaves simple, alternate, petiolate; petiole 1.5–2.5 cm long, pubescent, often twisted. Leaf blade narrowly lanceolate to subulate, 13–36 × 3–6 cm, papery; adaxial surface glabrous except for sparse pubescence along veins; abaxial surface sparsely appressed-pubescent; base rounded to truncate; apex acuminate; margin entire; venation pinnate, with one basal pair strongly ascending and connecting with lateral pairs, all converging toward the apex, giving a pseudo-triplinerved appearance; secondary veins 11–15 pairs. Inflorescences dimorphic: (1) axillary on young stems, peduncles 1–5 cm long, 2–8-noded, each node with a sessile bracteole, linear or lanceolate, 8–10 × 3–5 mm, rusty-villous; distal nodes fertile, each bearing a single flower; usually only the terminal node bearing one flower, occasionally the terminal and penultimate nodes both fertile; flowers opening basipetally. (2) cauliflorous on older stems: at persistent axillary nodes, fascicles of 8–15 cymules are produced. Cymules pedunculate; 8–15 cm long, 5–10-noded, densely rusty-villous, each node bearing a small sessile bracteole, linear or lanceolate, 8–10 × 3–5 mm. Flowers restricted to the distal 2–3 nodes, each 1-flowered (usually only the terminal node; occasionally also the penultimate or antepenultimate). Anthesis basipetal within each cymule. Pedicels 0.7–1.5 cm long, densely rusty-villous, often twisted. Perianth zygomorphic; abaxial surface yellowish-brown with purplish-red ridged venation, densely erect-villous. Utricle long-ovoid to cylindrical, 2–3 × 1–1.2 cm, constricted at ovary junction, dilated toward basal tube; adaxial side with a translucent white window at calyx–ovary junction; adaxial surface arachnoid-villous near basal tube, otherwise sparsely villous, base color vinaceous beneath indumentum. Basal tube 6.5–8.5 × 1.0–2.0 cm, reddish-brown, adaxially smooth, glabrous, waxy in texture. Upper tube 3–5 × 1.0–1.5 cm, inner surface yellow in proximal 1.5–2 cm, glabrous, waxy, distally black. Limb 3-lobed, lobes wholly connate; lower margin revolute upward, strongly incurved and connivent, auriform in frontal view; adaxial surface entirely black, muricate with coarse, fleshy, conical outgrowths; in natural revolute form limb region 5.0–6.0 × 7.5–10.0 cm, in pressed vouchers limb spreading to 7.5–10.0 × 7.5–10.0 cm, nearly circular. Throat elliptic, 1.8–3.0 × 1.5–2.0 cm, velvety black. Gynostemium 3-lobed, fleshy, 12–15 mm high, 3–6 mm diam., apex rounded. Anthers oblong, 7–10 mm long, extrorse. Ovary inferior, cylindric, slightly curved, with six adaxial ridges concealed by dense rusty indumentum. Fruit capsule not observed.

##### Paratypes.

China. • Guangxi: Liuzhou City, Rongshui Miao Autonomous County, Wangdong Township, Jiuwanshan Mountains, Yuyashan, 850–1180 m a.s.l., 26 Jun 1958, S.H.Chun *14711* (paratypes: IBSC! [0127615], IBK! [00014229]); • same county and township, Jiuwanshan Mountains, Lengshuiyao, 18 Jul 1958, S.H.Chun *15737* (paratypes: IBSC! [0127614, 0127619], IBK! [00014227], NAS! [00302195]). • Hechi City, Huanjiang County, Dongxing Township, Yangmeiao, along a ditch in broad-leaved forest, 1199 m a.s.l., 08 December 2012, Huanjiang County Survey Team *4512226121208009LY* (IBK! [00360056]).

##### Distribution and habitat.

*Aristolochia ferox* is currently known only from northern Guangxi Zhuang Autonomous Region, China, where it is largely distributed within or adjacent to the Jiuwanshan Mountain range. Three extant populations have been documented, all from Rongshui County, Liuzhou City, including one population in Anchui Township and two populations in Sirong Township. The species occurs on montane slopes composed of metamorphosed sandstone and siltstone at elevations of 500–1500 m a.s.l., and is commonly associated with riparian habitats along creeks. Two historical collections (S.H.Chun *14711*, *15737*) from Wangdong Township, Jiuwanshan Mountains, also within Rongshui County, indicate a former presence in this area (Suppl. material 1: figs S2, S3). In addition, a collection from Huanjiang County (Huanjiang County Survey Team *4512226121208009LY*), likewise within the Jiuwanshan Mountain range, further supports its historical distribution in this region.

##### Phenology.

The flowering season extends from June to November. Individual flowers remain open for up to two weeks. Fruits and seeds have not yet been observed.

##### Etymology.

The species epithet ferox (Latin: “fierce, wild, untamed”) refers to the wholly dark purple, muricate adaxial calyx limb that gives the perianth its formidable and aggressive appearance.

##### Vernacular name.

Chinese: 饕餮关木通 (*tāo tiè guān mù tōng*). Named for the resemblance of the flower’s frontal view to the gaping mouth of *Tāo Tiè* (饕餮), a mythical beast and cultural totem in Chinese folklore.

##### Usages and conservation status.

*Aristolochia ferox* is locally recognized as an ethnobotanical herb used in traditional practices for the treatment of snakebite and urologic ailments, with preparations made from dried underground rhizomes and old stems. According to our field surveys, these reported uses have not been clinically validated. Nevertheless, a local collection of this species for medicinal purposes was observed. Since its discovery, we have identified only three extant populations, each with fewer than five individuals, often with just one or two mature plants and no evidence of a stable population structure (i.e., no seedlings or young plants were found). Although individuals produce abundant flowers, the fruit set is extremely low, and capsules are not observed, suggesting possible reproductive difficulties in the remaining populations. Based on our field data, the Extent of Occurrence (EOO) is estimated at 104.196 km^2^ and the Area of Occupancy (AOO) at 12 km^2^, values that correspond to the Endangered (EN) thresholds under IUCN Red List Criteria B1ab(iii,v)+2ab(iii,v) (IUCN, 2012; IUCN Guidelines, 2024). However, given the extremely small number of mature individuals (<15 in total), the absence of any subpopulation exceeding 50 individuals, ongoing exploitation for folk medicine, and signs of reproductive limitation, we preliminarily assess *A. ferox* as Critically Endangered (CR) under both criteria C2a(i) and D, and strongly advocate for immediate measures to ensure its conservation.

## Discussion

For the typification of *Aristolochia ferox*, we designate three historical collections as paratypes. These specimens conform to the diagnostic characters of the new species and originate from the same locality context as the holotype. They include two collections from Rongshui County, Guangxi Zhuang Autonomous Region (26 June 1958, S.H.Chun *14711*; and 18 July 1958, S.H.Chun *15737*), both from the same administrative unit as the holotype locality, as well as a vegetative voucher specimen from a nearby locality in Hechi City, Huanjiang County (08 December 2012, Huanjiang Survey Team *4512226121208009LY*). All these specimens were historically identified as *A. championii*. However, previous studies and our recent field investigations indicate that *A. championii* is restricted to Hong Kong and the Lianhua Mountain range of eastern Guangdong Province, from Chaozhou to Huizhou (Merrill and Chun 1940; Liao et al. 2021). The two Rongshui collections include detailed original descriptions of floral morphology that closely match the diagnostic characters of *A. ferox* (Suppl. material 1: figs S2, S3). The Huanjiang County specimen, although originating from a different administrative unit, is located near the border of Rongshui County. Together with the two historical collections, all specimens originate from the Jiuwanshan National Nature Reserve. Based on geographic continuity and morphological consistency, we conclude that these specimens represent *A. ferox* and designate them here as paratypes.

Misidentification among *A. ferox* and morphologically similar congeners has been common, as evidenced by historical confusion between *A. ferox* and *A. championii*, as well as between *I. haimingii* and *A. championii* (Huang et al. 2022). To facilitate reliable identification, we provide a diagnostic key below to distinguish these taxa.

### Key to *Aristolochia ferox* and morphologically close species

**Table:** 

1	Leaves elliptic, ovate, or oblong	**2**
–	Leaves narrowly lanceolate to subulate	**3**
2	Perianth throat white to pale pinkish; adaxial limb dark purple with radiating white reticulate venation	***A. fangchi***
–	Perianth throat dark purple; adaxial limb dark purple, surface fully covered with minute papillae	***A. quangbinhensis***
3	Perianth limb bright yellow to greenish, with reddish-brown veins near the throat	***I. haimingii***
–	Perianth limb reddish-brown to black	**4**
4	Basal portion of calyx limb extended downward; adaxial surface colliculate only around throat; throat bright yellow with purplish-red flecks, semicircular to arch-shaped	***A. championii***
–	Basal portion of calyx limb strongly revolute, lower edge curving upward to meet upper edge and concealing throat in frontal view; adaxial surface entirely black, muricate with fleshy conical outgrowths; throat black, elliptic	***A. ferox* sp. nov**.

## Supplementary Material

XML Treatment for Aristolochia ferox

## Appendix 1

**Specimens of *Aristolochia fangchi* Examined**. CHINA. Guangdong Province: Zhaoqing City, Dinghu Mountain, Jilong Mountain, 22 April 1970, K.L.Shi *2* (IBSC! [0000646], [0000647], [0000648]); the same city, Dinghu Mountain, Longchuan Keng, 500 m a.s.l., 03 August 1963, K.C.Ting & K.L.Shi *823* (IBSC! [0000649], HITBC! [003990])

**Specimens of *Aristolochia championii* Examined**. CHINA. Hong Kong: J.G.Champion *155* (K! [000978969]), J.G.Champion *156* (K! [000820386]); the same city, Victoria Peak, 1880, s.coll. s.n. (K! [000978970]).

**Specimens of *Aristolochia haimingii* Examined**. CHINA. Guangxi Zhuang Autonomous Region: Laibin City, Jinxiu Yao Autonomous County, Jinxiu Town, protection station of Silver fir, 1136 m a.s.l., 28 July 2016, Y.S.Huang & Yan Liu *Y3143* (IBK!, GXMG!); the same individual plant, 23 July 2021, Y.S.Huang *DYS2021072301* (IBK!); Guilin City, Yanshan District, Botany Garden of Guilin, taken to cultivation from the type locality, 06 October 2020, Y.S.Huang *Y3310* (IBK!).

**Specimens of *Aristolochia quangbinhensis* Examined**. VIETNAM. Quang Binh Province: Minh Hoa district, Hoa Luong community, 380 m a.s.l., 03 April 2013, T.V.Do *39* (VNMN!, DR!); Quang Tri Province: Dakrong Commune (formerly Ta Long Commune, Dakrong District), Ta Lao Village, Dakrong N.R., remnants of primary evergreen broad-leaved forest on granite mountain, 450 m. a.s.l., 07 March 2024, V.T. Bui, M.T. Le, K.S. Nguyen, et al., *QT-DR 12* (HN!).

## Additional information

### Artificial Intelligence (AI) use

The authors accept full responsibility for the content of the manuscript, including the disclosure of any use of AI.

No AI tools were used in the preparation of this manuscript.

### Author ORCIDs

Y. Wang https://orcid.org/0009-0003-1339-1856

S. Liao https://orcid.org/0000-0002-3876-8002

S. Landrein https://orcid.org/0000-0003-0028-2450

K.S. Nguyen https://orcid.org/0000-0001-5171-4140

J.G. Onyenedum https://orcid.org/0000-0002-1047-9807

Z. Guo https://orcid.org/0009-0004-1817-9827

### Data availability

All of the data that support the findings of this study are available in the main text or Supplementary Information.

## Supplementary materials

Supplementary material 1 Additional figures Data type pdf Explanation note **fig. S1**. Floral morphological characters of *Isotrema haimingii* (**A–C**) and *A. fangchi* (**D–F**). **A, D** Flowers in situ; **B, E** Frontal views of flowers showing adaxial calyx limb characters; **C, F** Longitudinal dissections of flowers. Scale bars = 2.0 cm. A-care provided by Dr. Yusongh uang. **fig. S2**. Paratype voucher specimen of *A. ferox*, S.H.Chun *14711* (IBK [00014229]). **fig. S3**. Paratype voucher specimen of *A. ferox*, S.H.Chun *15737* (IBK [00014227]). https://binary.pensoft.net/file/1598516This dataset is made available under the Open Database License (http://opendatacommons.org/licenses/odbl/1.0/). The Open Database License (ODbL) is a license agreement intended to allow users to freely share, modify, and use this Dataset while maintaining this same freedom for others, provided that the original source and author(s) are credited. Yifan Wang, Shuai Liao, Sven Landrein, Khang Sinh Nguyen, Joyce G. Onyenedum, Zirui Guo
