# Supplementary material for: Aristolochia ferox, a new species of subg. Siphisia (Aristolochiaceae) from Guangxi, China
Source: PhytoKeys. 2026 Apr 17;273:171–83. doi: 10.3897/phytokeys.273.169468 (PMC13109732; doi:10.3897/phytokeys.273.169468)
Supplement: Supplementary material 1 — Additional figures [file phytokeys-273-171_article-169468__-s001.pdf]

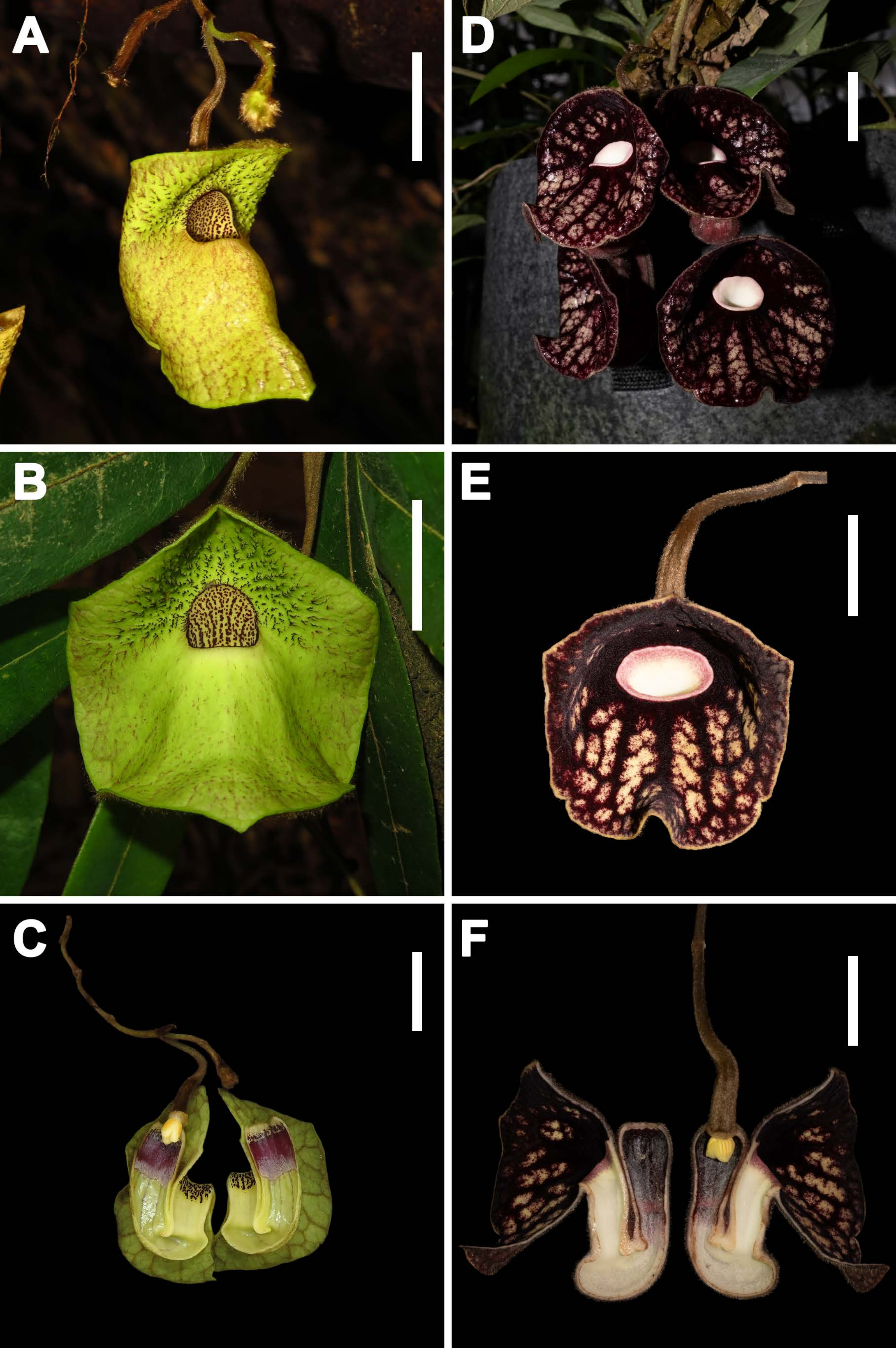
A
B
C
D
E
F

中國科学院華南植物研究所

采集記錄

标本号数：

采集人：陈少卿　采集号数：14711

采集日期：58 年 6 月 26 日

采集地点：广西大苗山县三防区[illegible]九万山 鱼尾山

海拔 1150–1250 米

環境：山地山谷密林常见散生

性狀：藤本木质缠绕　高　米；胸徑　厘米

叶 革质 正面绿色有斑点 背绿色 密被长毛

花 花被管 [illegible] 绿色 被绒毛 [illegible] 灰黑色 有凸起刺状

[illegible] 黑色 [illegible] 小 [illegible]

[illegible] 绿色有 [illegible] 三裂 花药 [illegible]

子房下位 六室

用途：花[illegible]

科名(号)：24

土名：　附記：

廣西省
KWANGSI

GUANGXI INSTITUTE OF BOTANY
IBK00014229

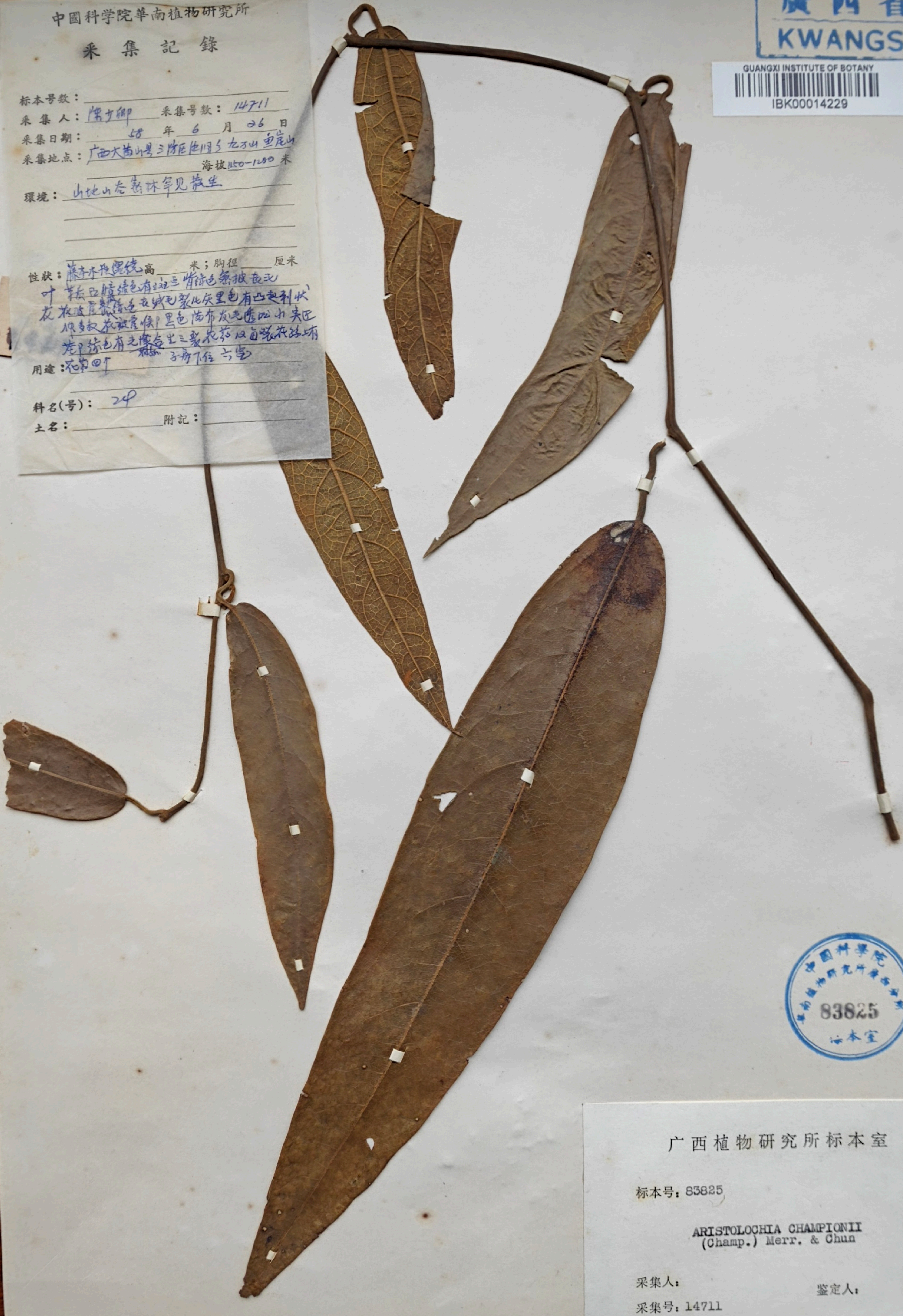

中國科學院 華南植物研究所標本室
83825

广西植物研究所标本室

标本号：83825

ARISTOLOCHIA CHAMPIONII (Champ.) Merr. & Chun

采集人：

鉴定人：

采集号：14711

GUANGXI INSTITUTE OF BOTANY
IBK00014227

廣西省
KWANGSI

中國科學院[illegible]

采集記錄

標本号數：　　采集号數：15737
采集人：陈少卿
采集日期：1958 年 7 月 18 日
采集地点：广西大苗山县三防区[illegible]九万山冷水[illegible]　海拔　米
環境：山谷，密林，肥土，少見，散生，
性狀：藤本，木质，高 6 米；胸徑　厘米
叶革质，正面绿色，背面绿色，密被白色长毛
花萼大似烟斗，[illegible]紫红色条纹，密被[illegible]，[illegible]
[illegible]
用途：
24
科名(号)：　　附記：
土名：

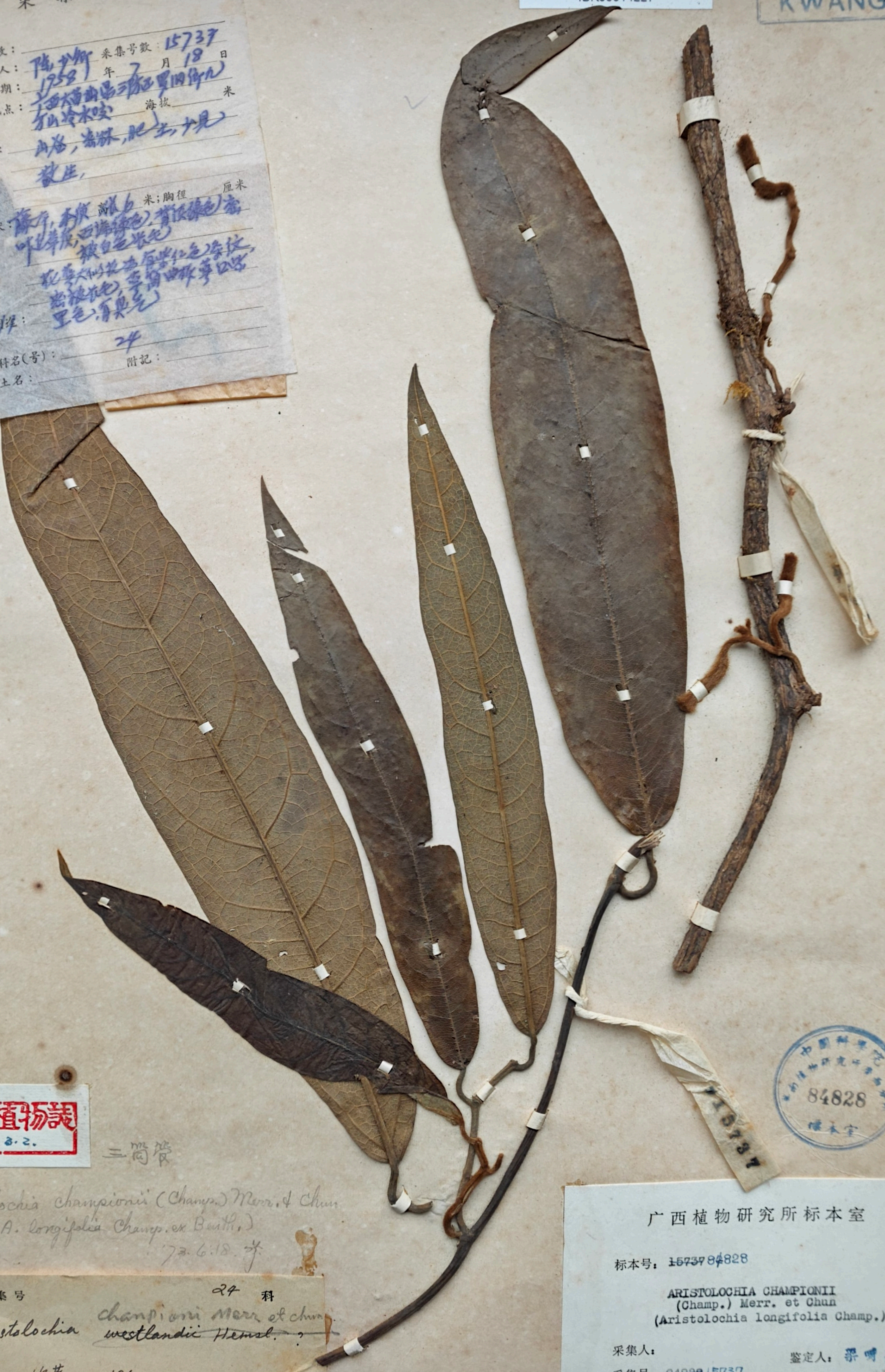

广西植物誌
[illegible] 74.3.2.

三筒管

Aristolochia championii (Champ.) Merr. & Chun
(A. longifolia Champ. ex Benth.)
'73.6.18. [illegible]

采集号　　24 科
Aristolochia championi Merr. et Chun
~~westlandii Hemsl.~~ ?
鑑定人：[illegible]　1962 年 12 月 15 日

中国科学院[illegible]
84828
标本室

15737

广西植物研究所标本室

标本号：~~15737~~ 84828

ARISTOLOCHIA CHAMPIONII
(Champ.) Merr. et Chun
(Aristolochia longifolia Champ.)

采集人：
鉴定人：梁畴芬
采集号：~~84828~~ 15737
